# Fluid-Phase Pinocytosis of Native Low Density Lipoprotein Promotes Murine M-CSF Differentiated Macrophage Foam Cell Formation

**DOI:** 10.1371/journal.pone.0058054

**Published:** 2013-03-11

**Authors:** Manoj K. Barthwal, Joshua J. Anzinger, Qing Xu, Thomas Bohnacker, Matthias P. Wymann, Howard S. Kruth

**Affiliations:** 1 Section of Experimental Atherosclerosis, National Heart, Lung, and Blood Institute, National Institutes of Health, Bethesda, Maryland, United States of America; 2 Department of Biomedicine, University of Basel, Basel, Switzerland; University of Padova, Italy

## Abstract

During atherosclerosis, low-density lipoprotein (LDL)-derived cholesterol accumulates in macrophages to form foam cells. Macrophage uptake of LDL promotes foam cell formation but the mechanism mediating this process is not clear. The present study investigates the mechanism of LDL uptake for macrophage colony-stimulating factor (M-CSF)-differentiated murine bone marrow-derived macrophages. LDL receptor-null (LDLR−/−) macrophages incubated with LDL showed non-saturable accumulation of cholesterol that did not down-regulate for the 24 h examined. Incubation of LDLR−/− macrophages with increasing concentrations of ^125^I-LDL showed non-saturable macrophage LDL uptake. A 20-fold excess of unlabeled LDL had no effect on ^125^I-LDL uptake by wild-type macrophages and genetic deletion of the macrophage scavenger receptors CD36 and SRA did not affect ^125^I-LDL uptake, showing that LDL uptake occurred by fluid-phase pinocytosis independently of receptors. Cholesterol accumulation was inhibited approximately 50% in wild-type and LDLR−/− mice treated with LY294002 or wortmannin, inhibitors of all classes of phosphoinositide 3-kinases (PI3K). Time-lapse, phase-contrast microscopy showed that macropinocytosis, an important fluid-phase uptake pathway in macrophages, was blocked almost completely by PI3K inhibition with wortmannin. Pharmacological inhibition of the class I PI3K isoforms alpha, beta, gamma or delta did not affect macrophage LDL-derived cholesterol accumulation or macropinocytosis. Furthermore, macrophages from mice expressing kinase-dead class I PI3K beta, gamma or delta isoforms showed no decrease in cholesterol accumulation or macropinocytosis when compared with wild-type macrophages. Thus, non-class I PI3K isoforms mediated macropinocytosis in these macrophages. Further characterization of the components necessary for LDL uptake, cholesterol accumulation, and macropinocytosis identified dynamin, microtubules, actin, and vacuolar type H(+)-ATPase as contributing to uptake. However, Pak1, Rac1, and Src-family kinases, which mediate fluid-phase pinocytosis in certain other cell types, were unnecessary. In conclusion, our findings provide evidence that targeting those components mediating macrophage macropinocytosis with inhibitors may be an effective strategy to limit macrophage accumulation of LDL-derived cholesterol in arteries.

## Introduction

Circulating low density lipoprotein (LDL) is the major carrier of cholesterol in the blood, and its level can predict the risk of developing atherosclerosis. Atherosclerosis progression involves accumulation of cholesterol in arterial macrophages to form foam cells. Since uptake of cholesterol-rich LDL by macrophages is a critical step for foam cell formation, targeting this pathway may be beneficial in the treatment of atherosclerosis.

Initially, investigators focused on modification of LDL as a mechanism to promote macrophage LDL uptake and foam cell formation [1]. The scavenger receptors CD36 and SRA were identified as important mediators of modified LDL uptake, and are thought to be relevant targets for preventing macrophage cholesterol accumulation [2]–[4]. However, CD36/SRA double knockout (KO) mice contain lipid-laden macrophages in vessel wall atherosclerotic plaques, suggesting that there may be additional mechanisms by which LDL can enter macrophages [5], [6].

Previous studies from our laboratory demonstrate that human macrophages take up native LDL by fluid-phase pinocytosis forming foam cells independently of receptors [7]–[12]. Macropinocytosis is a type of fluid-phase pinocytosis that occurs by vigorous actin-dependent membrane ruffling followed by ruffles fusing with the plasma membrane to form large vacuoles called macropinosomes [9], [13]. This type of fluid-phase pinocytosis can deliver large amounts of extracellular solute due to the large amount of fluid taken up within macropinosomes. Diverse growth factors, cytoskeletal proteins, signaling molecules such as GTPases (e.g., dynamin and rac1) and kinases (e.g., Pak1 and Src-family kinases) can modulate macropinocytosis variably in different cell types [14]–[16]. However, whether these factors regulate macrophage fluid-phase uptake of LDL has not been examined.

Macrophage colony-stimulating factor (M-CSF) is expressed in atherosclerotic lesions [17], and is necessary for monocyte as well as atherosclerosis development [18]–[21]. Furthermore, pharmacological inhibition of the M-CSF receptor, c-fms, with GW2580, or immunological inhibition with an anti-c-fms antibody both retard the progression of atherosclerosis [22], [23]. However, the anti-atherogenic effect of disrupting the M-CSF/c-fms receptor pathway is not completely explained by a decrease in monocytes, the precursor of macrophages [18]–[20]. This suggests that other macrophage functions mediated by M-CSF may contribute to the atherogenic effects of M-CSF. In this regard, M-CSF has been shown to stimulate macropinocytosis in murine M-CSF-differentiated bone marrow-derived macrophages [24]. Thus, it is of interest to determine whether M-CSF can stimulate mouse bone marrow-derived macrophage cholesterol accumulation due to fluid-phase uptake of LDL.

Pharmacological targeting of the phosphoinositide 3-kinase (PI3K) family of kinases with the pan-PI3K inhibitors wortmannin and LY294002 has previously been shown to inhibit macropinocytosis in murine M-CSF-differentiated bone marrow-derived macrophages [25]–[27]. However, the function of individual isoforms that mediate macropinocytosis has not been evaluated. The PI3K family consists of class I, II and III isoforms [28]. Class I isoforms are widely studied due to the availability of specific inhibitors and KO or kinase-dead knock-in (KI) mice [29]–[32]. However, specific class II and III PI3K isoform inhibitors and KO mice are not readily available. Although murine M-CSF-differentiated bone marrow-derived macrophages are known to display macropinocytosis [24]–[27], the molecular components mediating murine macrophage fluid-phase pinocytosis of LDL have not been characterized. Moreover, it is not known whether the PI3K family of kinases that mediate macropinocytosis in these cells also regulate LDL uptake and foam cell formation. Therefore, the present study was undertaken to investigate native LDL uptake by murine M-CSF-differentiated macrophages and to examine the molecular components that mediate this process. Our results demonstrate receptor-independent, fluid-phase pinocytosis of LDL by murine macrophages is dependent on non-class I PI3K isoforms. We also identify dynamin, actin, and microtubule cytoskeletal components as contributing to fluid-phase uptake of LDL. Furthermore, in contrast to their mediating macropinocytosis in other cell types [14]–[16], the signaling molecules Pak1, Rac1, and Src-family kinases did not mediate macropinocytosis in murine M-CSF-differentiated bone marrow-derived macrophages.

## Results

**Figure 1:**
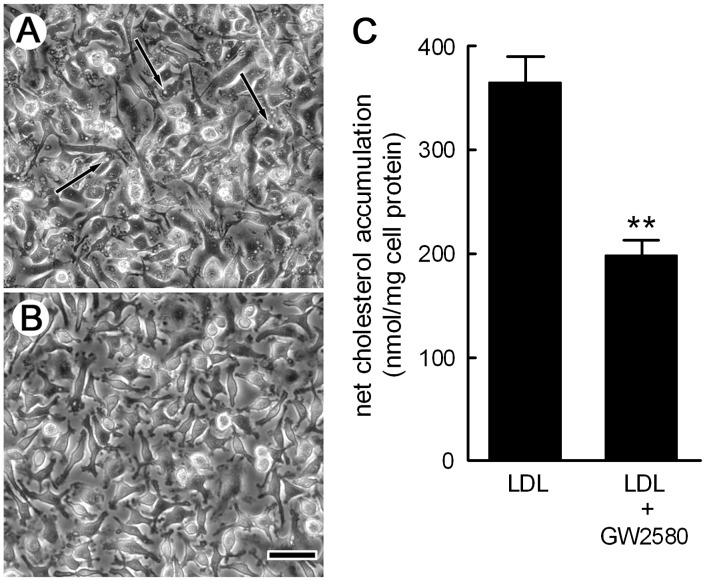
Macropinosome formation is M-CSF dependent. A and B. Wild-type macrophages were differentiated with M-CSF for 7 days and visualized by phase-contrast microscopy following the treatments described below. Macrophages differentiated with M-CSF were pretreated 30 min with DMSO drug vehicle (A), or 5 µM of cFMS (i.e., M-CSF receptor) tyrosine kinase inhibitor, GW2580 (B). Pretreatment was carried out without either serum or M-CSF. Withdrawal of M-CSF caused disappearance of the macrophage vacuoles. Subsequently, these macrophage cultures were treated 30 min with fresh serum-free medium containing M-CSF (50 ng/ml) without (A) or with GW2580 (B). Macrophages treated with M-CSF without GW2580 showed numerous vacuoles shown to be macropinosomes in Video S1. In contrast, there was complete inhibition of macropinosome formation when macrophage cultures were treated with GW2580 (also see Video S2). Scale bar in B = 75 µm and also applies to A. (C) Wild-type macrophages were incubated 24 h with 1 mg/ml LDL without or with 5 µM GW2580, and then cholesterol accumulation was assessed. Macrophages incubated without LDL had 111±3 nmol cholesterol/mg protein. ** = *p*<0.01.

Bone marrow from wild-type mice was differentiated with M-CSF to generate macrophages. These macrophages showed an elongated morphology and numerous cytoplasmic vacuoles that we determined were macropinosomes by time-lapse, phase-contrast microscopy (Figure 1A, Video S1). To eliminate the possibility that the LDL receptor mediates LDL uptake and cholesterol accumulation, we also generated macrophages from LDLR−/− mice. Wild-type macrophages were morphologically indistinguishable (including the presence of macropinosomes) from LDLR−/− macrophages (data not shown). The M-CSF receptor tyrosine kinase inhibitor GW2580 inhibited macropinocytosis and caused a loss of macropinosomes from the macrophages (Figure 1B, Video S2). Likewise, withdrawing M-CSF also inhibited macropinocytosis and caused a loss of macropinosomes (Videos S3 and S4), consistent with a previous report showing M-CSF is necessary for macropinocytosis in these macrophages [24]. Wild-type macrophages incubated 24 h with 1 mg/ml LDL and GW2580 (5 µM) showed a 46±4% reduction in net cholesterol accumulation compared with macrophages not treated with GW2580 (Figure 1C), thus implicating macropinocytosis in mediating about one-half of macrophage cholesterol accumulation. To determine if GW2580 was active for our experiments, we examined the effect of GW2580 on the M-CSF receptor by Western blotting (Figure S1). Tyrosine phosphorylation of the M-CSF receptor was similar for vehicle-treated and GW2580-treated macrophages. However, GW2580-treated lysates showed more M-CSF receptor compared with vehicle-treated lysates, indicating that GW2580 was active in some way in our experiments. We also incubated macrophages with LDL in the presence or absence of M-CSF to determine if M-CSF is a mediator of macrophage accumulation of LDL-derived cholesterol. Similar to GW2580, withdrawal of M-CSF from macrophages resulted in a decreased accumulation of LDL-derived cholesterol compared with macrophages incubated with M-CSF (Figure S2), indicating that M-CSF is a mediator of macrophage accumulation of LDL-derived cholesterol.

**Figure 2:**
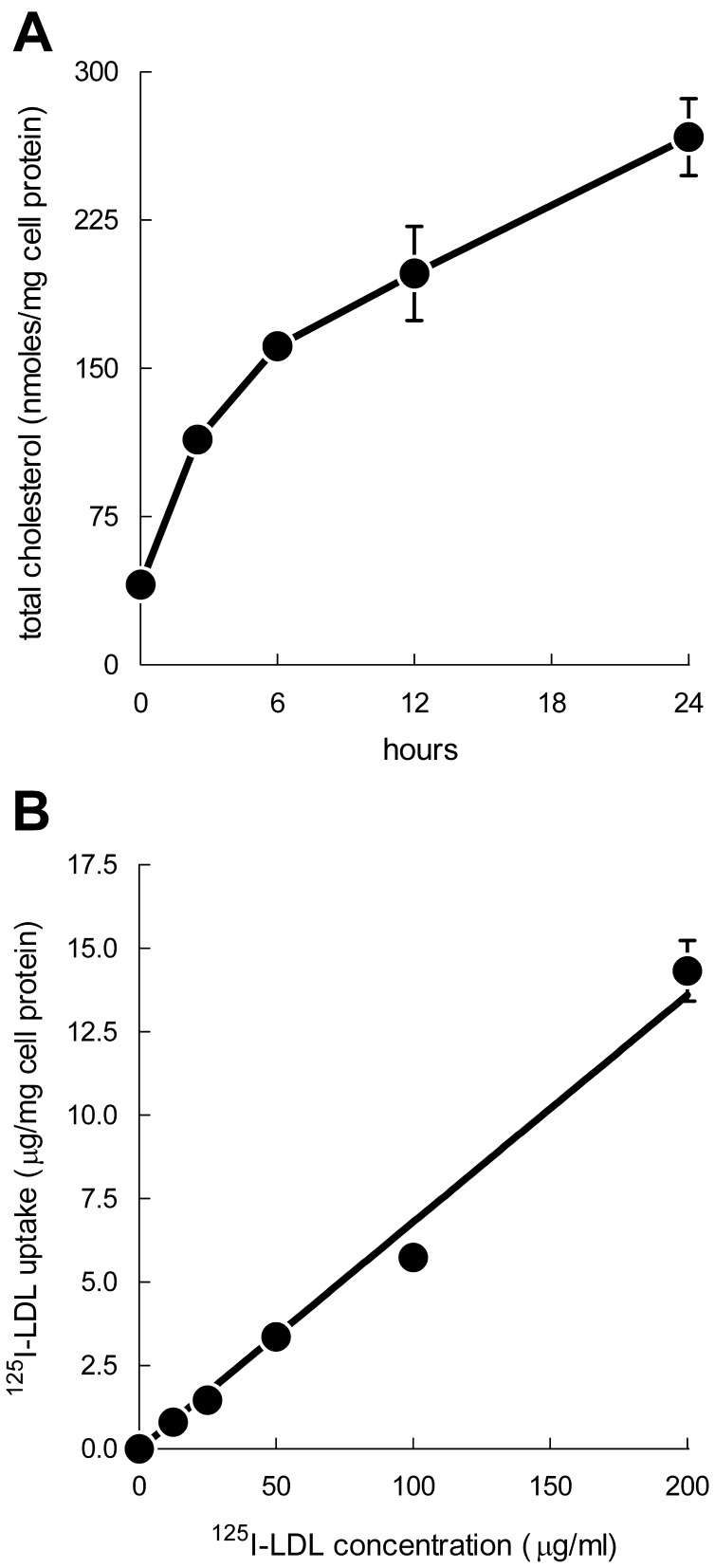
Macrophage uptake of ^125^I-LDL is non-saturable. A. LDLR−/− macrophages were incubated with 1 mg/ml of LDL for 0–24 h and cholesterol accumulation was then assessed. B. LDLR−/− macrophages were incubated 24 h with increasing concentrations of ^125^I-LDL, and then ^125^I-LDL uptake was assessed. Uptake values represent the sum of cell-associated and degraded ^125^I-LDL. The range of cell-associated and degraded ^125^I-LDL was 7–13% and 87–93%, respectively.

We next incubated LDLR−/− M-CSF-differentiated macrophages with increasing concentrations of LDL. Macrophages showed progressive accumulation of cholesterol (Figure S3A). The total cholesterol levels increased from 44±2 nmol/mg protein (13±4% esterified) without LDL addition to 518±9 nmol/mg protein (74±1% esterified) when macrophages were incubated with 4 mg/ml LDL. Staining of these cells with Oil Red O to detect neutral lipid revealed massive accumulation of lipid droplets in LDL-treated macrophages while no staining was observed in the absence of LDL (Figure S3, B and C). To determine whether macrophages incubated with LDL down-regulate cholesterol accumulation with time, we incubated macrophages with 1 mg/ml LDL up to 24 h and assessed cholesterol accumulation (Figure 2A). Macrophages showed a time-dependent increase in cholesterol accumulation showing that down-regulation of cholesterol accumulation did not occur within the incubation period examined. The total cholesterol level increased from 41±4 nmol/mg protein (13±0% esterified) at 0 h to 267±19 nmol/mg protein (72±2% esterified) at 24 h. These experiments show that M-CSF-differentiated macrophages accumulate LDL-derived cholesterol to form foam cells.

**Figure 3:**
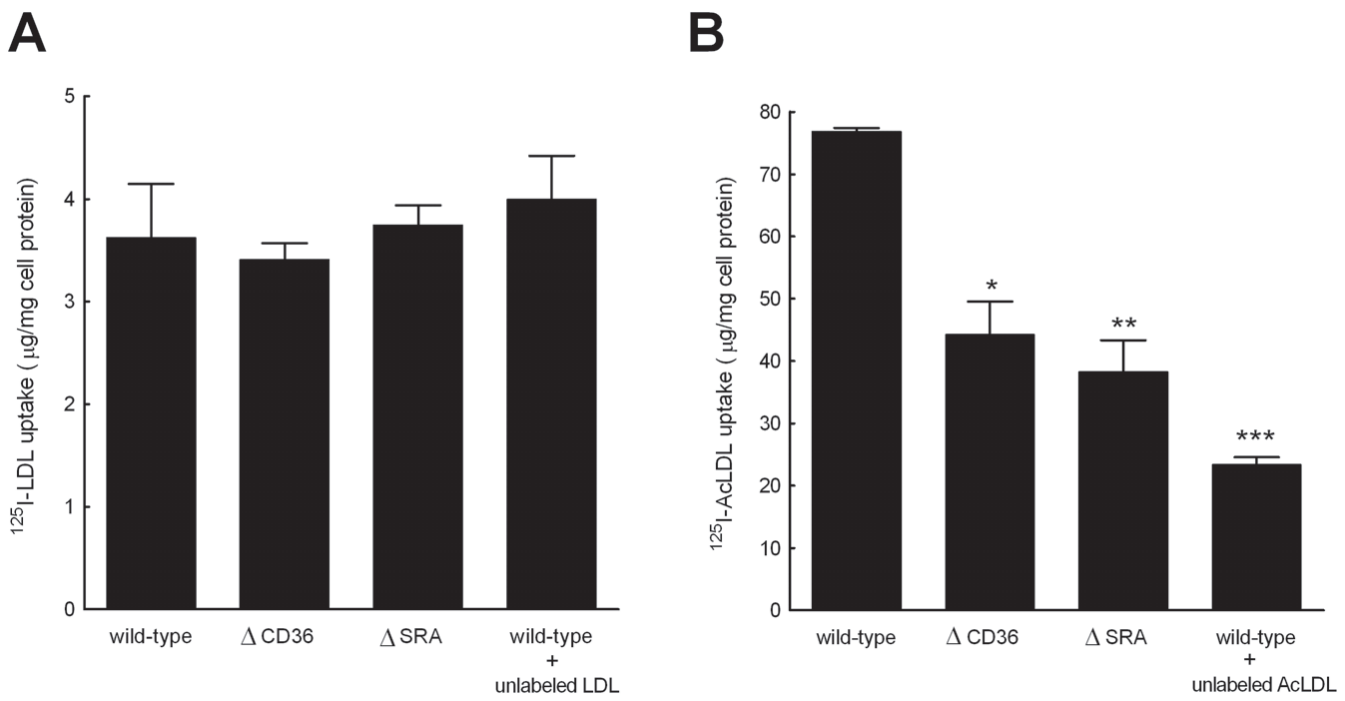
Fluid-phase pinocytosis mediates LDL uptake. (A) Wild-type macrophages were incubated 6 h with either 25 µg/ml ^125^I-LDL alone, or 25 µg/ml ^125^I-LDL and 500 µg/ml unlabeled LDL. CD36 KO macrophages (ΔCD36) and SRA KO macrophages (ΔSRA) macrophages were incubated 6 h with 25 µg/ml ^125^I-LDL alone. (B) Wild-type macrophages were incubated 6 h with either 25 µg/ml ^125^I-AcLDL alone, or 25 µg/ml ^125^I-AcLDL and 500 µg/ml unlabeled AcLDL. CD36 KO macrophages (ΔCD36) and SRA KO macrophages (ΔSRA) macrophages were incubated 6 h with 25 µg/ml ^125^I-AcLDL alone. Incubations were performed in serum-free medium containing 50 ng/ml M-CSF. Uptake values represent the sum of cell-associated and degraded ^125^I-labeled lipoprotein. The range of cell-associated and degraded ^125^I-LDL was 17–21% and 79–83%, respectively. The range of cell-associated and degraded ^125^I-AcLDL was 16–18% and 82–84%, respectively. ^125^I-LDL uptake was not competed with excess unlabeled LDL consistent with fluid-phase pinocytosis mediating uptake. Statistical tests compare each treatment group with wild-type macrophages incubated with 25 µg/ml ^125^I-AcLDL. * = *p*<0.05. ** = *p*<0.01. *** = *p*<0.001. There was no statistical difference between macrophage groups incubated with ^125^I-LDL.

Because previous reports from our laboratory show that fluid-phase pinocytosis mediates LDL accumulation in human macrophages [7]–[12], [33], we tested whether this also occurs for murine M-CSF-differentiated macrophages. When LDLR−/− macrophages were incubated with increasing concentrations of ^125^I-LDL (Figure 2B), a concentration-dependent, non-saturable uptake of native ^125^I-LDL was observed, consistent with fluid-phase pinocytosis rather than receptor-mediated endocytosis as mediating uptake of the LDL. To confirm that macrophage fluid-phase pinocytosis mediated LDL uptake, wild-type macrophages were incubated with ^125^I-LDL alone or with^ 125^I-LDL and a 20-fold excess of unlabeled LDL. Macrophage uptake of ^125^I-LDL was unaffected by the 20-fold excess of unlabeled LDL, confirming that fluid-phase pinocytosis mediated uptake of the ^125^I-LDL (Figure 3A). As a control, macrophages were also incubated with ^125^I-acetylated LDL (AcLDL) alone or with a 20-fold excess of unlabeled AcLDL. A 70% reduction of macrophage ^125^I-AcLDL uptake was observed when macrophages were incubated with ^125^I-AcLDL and a 20-fold excess of unlabeled Ac-LDL compared with macrophages incubated with ^125^I-AcLDL alone. This finding of competitive inhibition was consistent with the known scavenger receptor-mediated uptake of this modified form of LDL (Figure 3B).

The scavenger receptors CD36 and SRA are believed to be necessary for macrophage uptake of LDL [2]–[4]. Our experiments strongly suggest that these receptors are not necessary for macrophage uptake of LDL. To directly determine if CD36 and SRA mediate LDL uptake, macrophages from wild-type, CD36-null (CD36−/−), and SRA-null (SRA−/−) mice were generated and then incubated with 25 µg/ml native ^125^I-LDL, or as a control 25 µg/ml ^125^I-AcLDL, a modified lipoprotein known to bind to CD36 and SRA [4]. CD36−/− and SRA−/− macrophages showed similar levels of ^125^I-LDL uptake compared with wild-type macrophages (Figure 3A). We also observed similar levels of ^125^I-LDL uptake for wild-type, CD36−/−, and SRA−/− macrophages incubated with 200 µg/ml ^125^I-LDL (Figure S4). As expected, ^125^I-AcLDL uptake was significantly lower for CD36−/− or SRA−/− macrophages compared with wild-type macrophages (Figure 3B). These results conclusively demonstrate that the scavenger receptors CD36 and SRA are not necessary for macrophage uptake of LDL.

Because previous studies suggest that the PI3K family of kinases function in mediating fluid-phase macropinocytosis [25], [26], we tested whether this family of kinases is involved in LDL-derived cholesterol accumulation in murine M-CSF-differentiated macrophages. An approximate 50% decrease in net cholesterol accumulation was observed for LDLR−/− macrophages incubated with LDL and treated with the pan-PI3K inhibitors, LY294002 or wortmannin (Table 1). To test whether inhibition of cholesterol accumulation was due to inhibition of LDL uptake, we assessed ^125^I-LDL uptake by LDLR−/− macrophages. An approximate 50% inhibition of LDL uptake was observed for LDLR−/− macrophages treated with LY294002 or wortmannin (Table 1), and thus accounted for the inhibition of net cholesterol accumulation.

**Table 1:** The effect of PI3K inhibitors and cytochalasin D on LDL uptake and net cholesterol accumulation in LDLR −/− macrophages.

	Inhibitor		
Measurement	LY294002	Wortmannin	Cytochalasin D
Percent inhibition of net cholesterol accumulation	49±3***	50±7**	69±9***
Percent inhibition of ^125^I-LDL uptake	52±7***	56±8***	65±1***

LDLR−/− bone marrow-derived macrophages were pretreated 1 h with either drug vehicle, 50 µM LY294002, 100 nm wortmannin, or 4 µg/ml cytochalasin D. For cholesterol accumulation, macrophages then were incubated with 1 mg/ml LDL and inhibitor for 24 h. For ^125^I-LDL uptake, macrophages then were incubated 5 h with 200 µg/ml ^125^I-LDL and inhibitor. All incubations were performed in serum-free medium containing 50 ng/ml M-CSF. The percent inhibition of net cholesterol accumulation compares macrophages treated with LDL alone and LDL with inhibitor after basal cholesterol values were subtracted from each. The percent inhibition of ^125^I-LDL uptake compares macrophages treated with ^125^I-LDL alone and ^125^I-LDL with inhibitor. ** = *p*<0.01. *** = *p*<0.001. All inhibitors showed almost complete inhibition of macropinosome formation as assessed by phase-microscopy. For LY294002 and wortmannin experiments, control macrophages incubated with LDL or ^125^I-LDL alone showed net cholesterol accumulation and ^125^I-LDL uptake values of 226±8 nmole/mg cell protein and 2.7±0.1 µg/mg cell protein, respectively. For cytochalasin D experiments, control macrophages incubated with LDL or ^125^I-LDL alone showed net cholesterol accumulation and ^125^I-LDL uptake values of 230±2 nmole/mg cell protein and 3.4±0.1 µg/mg cell protein, respectively. The range of cell-associated and degraded ^125^I-LDL for all treatments was 19–33% and 67–81%, respectively.

Fluid-phase pinocytosis mediated by macropinocytosis is an actin-dependent process. To examine whether LDLR−/− macrophage uptake of LDL is dependent on actin polymerization, we assessed net cholesterol accumulation and LDL uptake in the presence of the actin polymerization inhibitor, cytochalasin D (Table 1). A 69% inhibition of macrophage net cholesterol accumulation was observed (Table 1). Similarly, a 65% inhibition in ^125^I-LDL uptake was observed, indicating that inhibition of net cholesterol accumulation was due to inhibition of macrophage uptake of LDL. Macropinocytosis assessed by phase-contrast microscopy was inhibited by LY294002, wortmannin, and cytochalasin D (Figure S5, A–C and data not shown), suggesting that these inhibitors affect cholesterol accumulation in the LDLR−/− cells by regulating fluid-phase macropinocytosis of native LDL.

**Figure 4:**
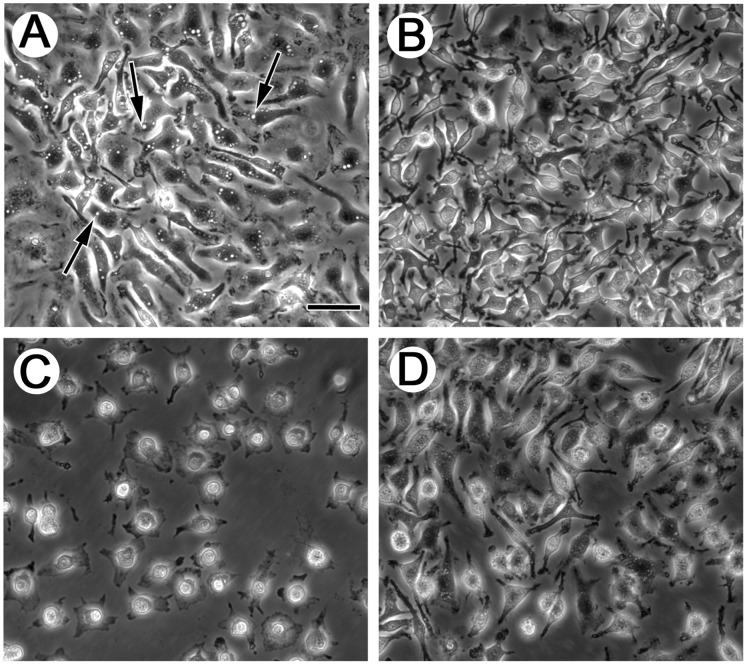
Macropinocytosis is inhibited by PI3K and actin polymerization inhibitors. Wild-type macrophages differentiated with M-CSF were pretreated 30 min with the drugs indicated below but without serum and M-CSF. Then, macrophages were treated 30 min with fresh serum-free medium containing M-CSF (50 ng/ml) in the presence of either DMSO vehicle (A), 100 nM wortmannin (B), 4 µg/ml cytochalasin D (C), or 50 µM LY294002 (D), and then examined by phase-contrast microscopy. Arrows indicate macropinosomes. Scale bar in A = 75 µm and applies to all. (Also see Videos S5, S6, S7, S8).

**Figure 5:**
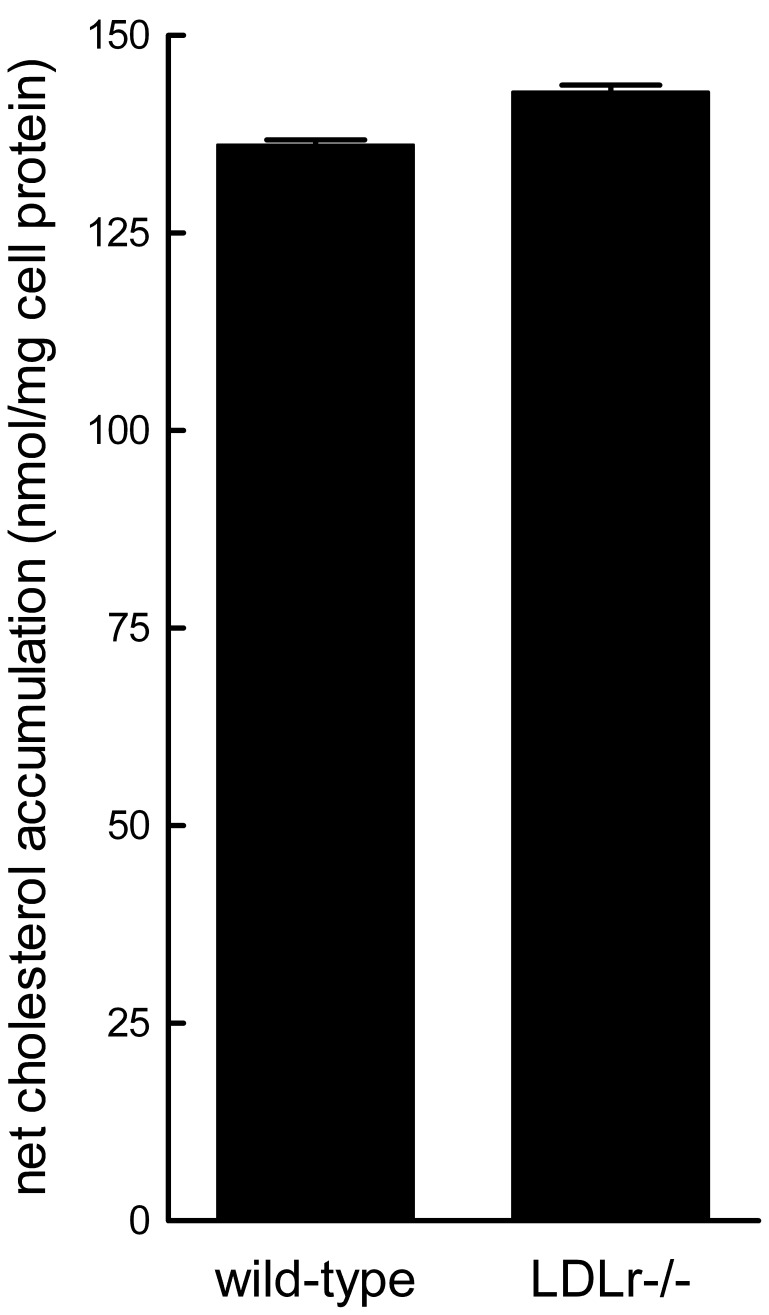
Wild-type and LDL−/− macrophages incubated with LDL accumulate similar levels of cholesterol. Wild-type and LDLR−/− macrophages were incubated with 1 mg/ml LDL for 24 h and then total cholesterol accumulation was assessed. The baseline cholesterol levels for wild-type and LDLR−/− macrophages were 107±10 nmol cholesterol/mg protein and 122±9 nmol cholesterol/mg protein, respectively.

Similar to LDLR−/− macrophages, wild-type macrophages treated with LY294002, wortmannin, or cytochalasin D showed a 54%, 59% and 64% inhibition, respectively, of net cholesterol accumulation (Table 2). These drugs inhibited macrophage macropinocytosis in these wild-type macrophages (Figure 4, A–D; Videos S5, S6, S7, S8) indicating that fluid-phase macropinocytosis of LDL was responsible for about one-half of the macrophage fluid-phase uptake of LDL, similar to what was observed for LDLR−/− macrophages. Because wild-type and LDLR−/− macrophages showed similar inhibition of LDL uptake and LDL-derived cholesterol accumulation, these results show that the two types of macrophages are phenotypically similar with respect to macropinocytic uptake of LDL. Consistent with this conclusion, net cholesterol accumulation during a 24 h incubation with 1 mg/ml LDL for wild-type and LDLR−/− macrophages was similar, 136±1 nmol/mg cell protein and 143±1 nmol/mg cell protein, respectively (Figure 5).

**Table 2:** The effect of PI3K inhibitors and cytochalasin D on net cholesterol accumulation in wild-type macrophages.

	Inhibitor		
Measurement	LY294002	Wortmannin	Cytochalasin D
Percent inhibition of net cholesterol accumulation	54±4***	56±11***	64±0***

Wild-type bone marrow-derived macrophages were pretreated 1 h with either drug vehicle, 50 µM LY294002, 100 nm wortmannin, or 4 µg/ml cytochalasin D. Macrophages then were incubated with 1 mg/ml LDL and inhibitor for 6 h. Incubations were performed in serum-free medium containing 50 ng/ml M-CSF. The percent inhibition of net cholesterol accumulation compares macrophages treated with LDL alone and LDL with inhibitor after basal cholesterol values were subtracted from each. ** = *p*<0.01. *** = *p*<0.001. All inhibitors almost completely inhibited macropinosome formation as assessed by phase-microscopy. For the LY294002 experiment, control macrophages incubated with LDL alone showed net cholesterol accumulation of 31±2 nmole/mg cell protein. For the wortmannin and cytochalasin D experiments, control macrophages incubated with LDL alone showed net cholesterol accumulation of 78±2 nmole/mg cell protein.

**Figure 6:**
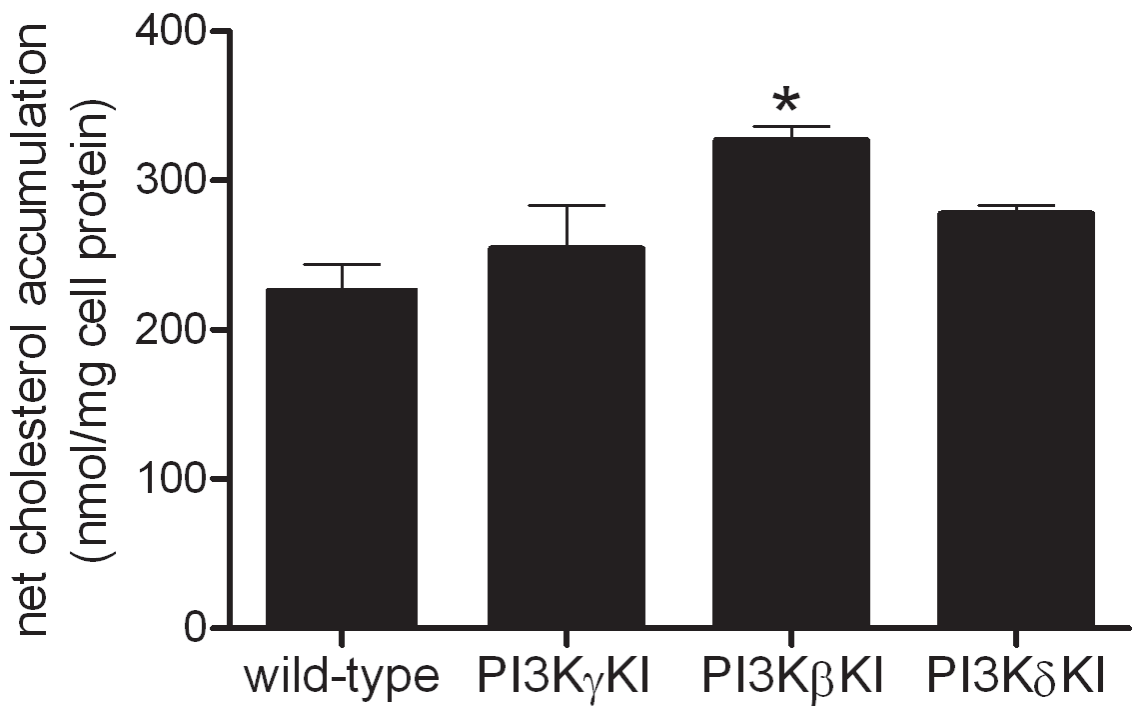
LDL-derived cholesterol accumulation occurs independently of class I PI3K isoforms. Cholesterol accumulation after 24 h-incubation without or with 1 mg/ml LDL was assessed in M-CSF differentiated macrophages cultured from wild-type, PI3Kγ-KI, PI3Kβ-KI, and PI3Kδ-KI mice.

Having observed that PI3K inhibition showed significant inhibition of LDL uptake, net cholesterol accumulation, and macropinocytosis, we examined the function of individual PI3K isoforms with respect to macropinocytosis and macrophage cholesterol accumulation. We assessed LDL-derived cholesterol accumulation by PI3K kinase-dead KI macrophages for the class I PI3K isoforms beta, delta, gamma, or by wild-type mice treated with class I PI3K isoform-specific small molecule inhibitors. No morphological differences including the presence of macropinosomes were observed comparing wild-type and the PI3K kinase-dead KI macrophages (data not shown). All PI3K kinase-dead KI macrophages showed an elongated morphology and macropinosomes, similar to wild-type macrophages. No significant difference in LDL-derived net cholesterol accumulation was observed comparing macrophages derived from wild-type, PI3K gamma kinase-dead KI, and PI3K delta kinase-dead KI mice (Figure 6). Surprisingly, an increase in net cholesterol accumulation was observed for PI3K beta kinase-dead KI macrophages incubated with LDL compared with wild-type macrophages (Figure 6). LDL-derived net cholesterol accumulation was also assessed for wild-type macrophages incubated with LDL without or with inhibitors of PI3K alpha, beta, gamma or delta isoforms. Macrophage net cholesterol accumulation and macropinocytosis were unaffected by these PI3K isoform-specific inhibitors (Table 3). These results suggest that a PI3K isoform other than class I PI3K mediates murine M-CSF macrophage macropinocytosis of LDL.

**Table 3:** The effect of possible inhibitors of fluid-phase pinocytosis on LDL uptake and net cholesterol accumulation in wild-type M-CSF-differentiated macrophages.

Inhibitor (Concentration)	Target	Percent inhibition of net cholesterol accumulation	Percent inhibition of^125^I-LDL uptake	Inhibition ofmacropinocytosis
Nocodazole(10 µM)	microtubules	19±3**(78±2)	57±3**(10.6±0.2)	+
Bafilomycin(500 nM)	vacuolar type H(+)-ATPase	56±11**(117±10)	67±2***(10.6±0.2)	+
Dynasore (80 µM)	dynamin GTPase	77±2***(39±2)	67±4***(10.6±0.2)	+
Dynamin peptide inhibitor(20 µM)	dynamin GTPase	37±4*(132±12)	ND	+
PP2(20 µM)	Src-family kinases	13±2(88±4)	7±3(10.6±0.2)	–
PI103(1 µM)	PI3K Class I isoforms and Class II beta isoform	−3±8(31±2)	ND	–
PAK18(10 µM)	Pak1 kinase	18±8(117±10)	ND	–
NSC23766(100 µM)	Rac1 GTPase	18±9(117±10)	ND	–
Y-27632(40 µM)	Rho-associated protein kinases	19±10(117±10)	ND	–
PI3K alpha inhibitor-4(3 µM)	PI3K alpha	0±17(103±5)	ND	–
TGX-221(1 µM)	PI3K beta	12±6(31±2)	ND	–
IC87114(1 µM)	PI3K delta	12±4(31±2)	ND	–
AS605240(1 µM)	PI3K gamma	−10±2(31±2)	ND	–

Wild-type bone marrow-derived macrophages were pretreated 1 h with drug vehicle or the indicated drug. For cholesterol accumulation, macrophages then were incubated with 1 mg/ml LDL and inhibitor for 6 h. For ^125^I-LDL uptake, macrophages then were incubated with 200 µg/ml ^125^I-LDL and inhibitor for 6 h. All incubations were performed in serum-free medium containing 50 ng/ml M-CSF. The percent inhibition of net cholesterol accumulation compares macrophages treated with LDL alone and LDL with inhibitor (except the experiment that compares macrophages treated with LDL and dynamin peptide inhibitor with LDL and control peptide) after basal cholesterol values were subtracted from each. The percent inhibition of ^125^I-LDL uptake compares macrophages treated with ^125^I-LDL alone and ^125^I-LDL with inhibitor. Control values for macrophages incubated with LDL alone or ^125^I-LDL alone are indicated in parentheses. For cholesterol accumulation, control values are expressed as nmol net cholesterol accumulation/mg cell protein. For ^125^I-LDL uptake, control values are expressed as µg ^125^I-LDL uptake/mg cell protein. The range of cell-associated and degraded ^125^I-LDL for all treatments except bafilomycin A1-treated macrophages was 15–19% and 82–86%, respectively. Cell-associated and degraded ^125^I-LDL for bafilomycin A1-treated macrophages was 91% and 9%, respectively. * = *p*<0.05. ** = *p*<0.01. *** = *p*<0.001. ND = not determined. “+” indicates a decrease in the number of macropinosomes and “−” indicates no effect on macropinosomes. “−” in percent inhibition columns indicates stimulation rather than inhibition.

**Figure 7:**
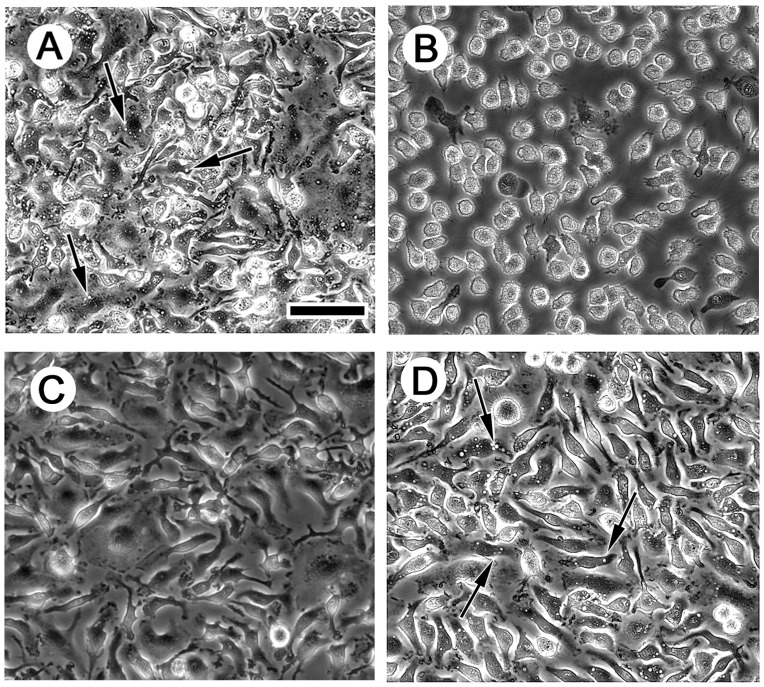
Macrophage macropinosome formation is dependent on dynamin and vacuolar type H(+)-ATPase. Wild-type M-CSF differentiated macrophages were pretreated 30 min with the indicated drugs but without serum and M-CSF. Subsequently, macrophages cultures were treated 30 min with fresh serum-free medium containing M-CSF (50 ng/ml) and either drug vehicle (A), 80 µM dynasore (B), 500 nM bafilomycin A1 (C), or 100 µM NSC23766 (D). Then, macropinosome formation was assessed by phase-contrast microscopy. Scale bar in A = 75 µm and applies to all. (Also see Videos S9, S10, S11, S12, S13, S14).

Because the molecular components mediating macropinocytosis may vary from one cell type to another [14]–[16], on the basis of published literature we tested several potential signaling molecules that may modulate murine M-CSF-differentiated macrophage macropinocytosis of LDL (Table 3). To examine the potential function of small GTPases and associated kinases, we monitored net cholesterol accumulation in the presence of LDL and Rac1 or Rho-associated kinase inhibitors (NSC23766 and Y-27632, respectively). Neither of these inhibitors significantly affected net cholesterol accumulation (Table 3). Consistent with the lack of effect of these GTPase inhibitors on macrophage cholesterol accumulation, wild-type macrophage macropinocytosis was also unaffected by the presence of these inhibitors (Table 3; Figure 7, A and D; Videos S9, S12, and S14). In contrast, the dynamin inhibitor, dynasore, inhibited net cholesterol accumulation and ^125^I-LDL uptake approximately 70% (Table 3) and also macropinocytosis (Figure 7B and Video S10). A dynamin specific peptide inhibitor also inhibited macropinocytosis (Videos S15 and S16) and LDL-derived cholesterol accumulation (Table 3), confirming that dynamin is a mediator of macropinocytosis. Pak1 kinase and Src-family kinases have been shown to modulate fluid-phase pinocytosis for various cells [14]–[16], but inhibitors of these kinases did not alter macrophage net cholesterol accumulation (Table 3) or macropinocytosis (data not shown).

As Rac1 and Src have been shown to mediate fluid-phase macropinocytosis [34], [35], we sought to confirm that the inhibitors used to target these molecules were active. Untreated macrophages showed GTP-bound Rac1 (i.e., activated Rac1), whereas macrophages treated with the Rac1 inhibitor NSC23766 showed almost no detectable GTP-bound Rac1 as determined by Western blot, confirming the effectiveness of this Rac1 inhibitor (Figure S6A). Similarly, untreated macrophages contained tyrosine phosphorylated Src-family kinase (i.e., activated Src-family kinase), whereas macrophages treated with the Src-family kinase inhibitor PP2 showed no Src-family kinase tyrosine phosphorylation, confirming the effectiveness of this Src-family kinase inhibitor (Figure S6B). These results confirm that the Rac1 inhibitor NSC23766 and the Src-family kinase inhibitor PP2 are active in macrophages, and confirm that Rac1 and Src-family kinases do not mediate macrophage fluid-phase macropinocytosis of LDL.

To assess the function of the structural component of microtubules, tubulin, in macrophage LDL uptake and cholesterol accumulation, macrophages were incubated with LDL without or with nocodazole, a microtubule inhibitor. An approximate 20% reduction of net cholesterol accumulation and 60% reduction of ^125^I-LDL uptake was observed in the presence of nocodazole compared with untreated macrophages (Table 3). Nocodazole also seemed to decrease macropinocytosis somewhat, but this was difficult to assess because nocodazole caused the macrophages to narrow (Video S13). Bafilomycin A1, a vacuolar H+-ATPase inhibitor, inhibited net cholesterol accumulation approximately 55% and ^125^I-LDL uptake approximately 65% for wild-type macrophages (Table 3). Macrophage macropinocytosis was also completely inhibited by bafilomycin A1 compared with untreated macrophages (Figure 7C; Video S11).

## Discussion

In the present study, we demonstrate for the first time that murine M-CSF-differentiated bone marrow-derived macrophages take up native LDL by fluid-phase pinocytosis to form foam cells. We identify macropinocytosis as a major mechanism of LDL uptake in these macrophages. Our results showing a linear and non-saturable uptake of LDL strongly suggests a receptor-independent mechanism mediating LDL uptake and cholesterol accumulation. The LDL receptor had no effect on macrophage uptake of LDL and cholesterol accumulation, because wild-type and LDLR−/− macrophages showed similar levels of cholesterol accumulation. These results rule out the possibility that the LDL receptor mediated macrophage LDL uptake, and is consistent with the fact that we did not upregulate the LDL receptor through cholesterol removal from the medium prior to incubations with LDL. We confirmed that receptors do not mediate LDL uptake because wild-type macrophages incubated with ^125^I-LDL and a 20-fold excess of unlabeled LDL showed similar uptake compared with macrophages incubated with ^125^I-LDL only. If receptors mediated LDL uptake, it would be expected that unlabeled LDL would compete with ^125^I-LDL for receptor binding. We observed no effect of unlabeled competitor for macrophage uptake of ^125^I-LDL. Additional experiments showed that the scavenger receptors CD36 and SRA do not mediate macrophage uptake of LDL because genetic deletion of these receptors did not affect LDL uptake. Macrophages incubated with ^125^I-AcLDL and a 20-fold excess of unlabeled AcLDL showed an approximate 70% reduction in ^125^I-AcLDL uptake compared with macrophages incubated with ^125^I-AcLDL only, as expected. Because uptake of AcLDL is mediated by scavenger receptors [1], its uptake by the macrophages could be competed. ^125^I-AcLDL uptake by CD36−/− or SRA−/− macrophages was significantly less than wild-type macrophages, demonstrating the important role of these receptors in mediating modified LDL uptake by macrophages.

The intima of the vessel wall (where atherosclerotic plaques develop) contains high concentrations of LDL (0.7–2.7 mg/ml) [36]–[38]. Thus, our experiments were designed to demonstrate foam cell formation with these physiological concentrations of LDL. We previously showed that macrophage fluid-phase pinocytosis occurs at high levels within atherosclerotic plaques [8]. Thus, the results of the current study adds to our previous studies [7]–[12] indicating that plaque macrophages can become foam cells by fluid-phase pinocytosis of LDL within the vessel wall. Studies of atherosclerosis development in scavenger receptor KO mice suggest that an alternative mechanism for macrophage foam cell formation exists. This is because mice lacking the scavenger receptors that mediate macrophage uptake of modified LDL in vitro, nevertheless, show macrophage foam cell formation in vivo [5], [6]. Our results provide a plausible mechanism for macrophage foam cell formation mediated by fluid-phase pinocytosis of LDL in mice with or without those scavenger receptors that mediate uptake of modified LDL.

In an earlier study [24] and confirmed in our current study, withdrawal of M-CSF from the culture medium of murine M-CSF-differentiated bone marrow-derived macrophages completely inhibited macropinocytosis, however fluid-phase pinocytosis was inhibited only 50%. Also, while M-CSF-stimulated macropinocytosis was completely inhibited by GW2580, an inhibitor of the M-CSF receptor tyrosine kinase activity, macrophage accumulation of cholesterol was inhibited only about 50%. Furthermore, wortmannin completely inhibited macropinocytosis, but inhibited fluid-phase uptake of LDL by only 50%. All these observations suggest that fluid-phase micropinocytosis mediated the remaining 50% of solute uptake by murine M-CSF-differentiated macrophages. This finding is similar to what we previously observed for human M-CSF-differentiated monocyte-derived macrophages that show approximately 50% of LDL uptake occurring by macropinocytosis and 50% occurring by micropinocytosis [7].

Although the M-CSF receptor is the only known receptor for M-CSF, and withdrawal of M-CSF inhibited both macropinocytosis and cholesterol accumulation, we cannot conclude that M-CSF signaling directly mediates macropinocytosis and cholesterol accumulation in these macrophages. This is because the M-CSF receptor tyrosine kinase inhibitor, GW2580, did not decrease the absolute level of M-CSF receptor tyrosine kinase activity. Possibly, GW2580 is inhibiting some other kinase that mediates macropinocytosis in these macrophages. Regardless, we show in this study that GW2580 inhibits macropinocytosis, which could explain why this inhibitor impedes the development of atherosclerosis [23].

In the present study, we observed inhibition of macrophage uptake of LDL, cholesterol accumulation, and macropinocytosis by dynasore. Dynasore also may have inhibited fluid-phase micropinocytosis because the total inhibition of LDL uptake was substantially greater than the amount of uptake inhibited by the macropinocytosis inhibitors, LY294002 and wortmannin (discussed below). In this regard, dynamin 2 mediates fluid-phase micropinocytosis in epithelial cells [39]. We also observed inhibition of macropinocytosis with a dynamin-specific peptide inhibitor, confirming that dynamin is a mediator of macropinocytosis. However, inhibition of macrophage LDL-derived cholesterol accumulation with dynamin-specific peptide inhibitor was less than that observed with dynasore. It is possible that this difference is due to the different inhibition mechanisms of these inhibitors (dynasore is a non-competitive inhibitor, whereas the dynamin-specific peptide inhibitor is a competitive inhibitor). Regardless of the difference observed for macrophage cholesterol accumulation, our data shows that dynamin is a mediator of macropinocytosis in murine M-CSF-differentiated macrophages. Interestingly, a recent study has shown that dynasore affects downstream signaling mediators of M-CSF receptor signaling [40], suggesting that dynasore, similar to GW2580, inhibits macropinocytosis by targeting M-CSF signaling. Whether dynamin inhibits macropinocytosis in non-macrophages is controversial (discussed in [39]). Because the development of atherosclerotic lesions is inhibited in atherosclerosis-prone mice fed dynasore, our results suggest that inhibition of macrophage fluid-phase pinocytosis of LDL with dynasore may contribute to dynasore inhibition of atherosclerosis in mice [41].

The molecular components mediating macropinocytosis by human M-CSF-differentiated monocyte-derived macrophages is different than that of murine M-CSF-differentiated bone marrow-derived macrophages, because macropinocytosis in mouse macrophages was blocked by bafilomycin, an inhibitor of vacuolar type H(+)-ATPase, while this was not the case for human macrophages in our earlier study [11]. Also, macropinocytosis by the human M-CSF-differentiated macrophages continued following M-CSF withdrawal, while macropinocytosis by the murine M-CSF-differentiated macrophages was M-CSF dependent.

Macropinocytosis is induced by transfection of Rac and Pak1 signaling molecules in non-macrophage cell types [35], [42], [43], and these factors have been implicated in the signaling that mediates macropinocytosis induced by PDGF and EGF growth factors in non-macrophage cells [42], [43]. Also, pharmacologic inhibition of Rho-associated kinase has been reported to disrupt macropinocytosis [44]. However, we found that these signaling molecules did not mediate M-CSF-induced macropinocytosis by mouse macrophages. Similarly, although certain Src-family kinases can induce macropinocytosis in transfected cells [34], [45], [46], murine macrophage macropinocytosis was not Src-family kinase-dependent, similar to what we previously observed in human M-CSF-differentiated monocyte-derived macrophages [7].

Also, macropinocytosis in human M-CSF-differentiated monocyte-derived macrophages is constitutive and does not depend on the presence of M-CSF, whereas as discussed above, murine macrophage macropinocytosis did depend on the presence of M-CSF. It is possible that these differences represent a species difference in the regulation of macropinocytosis, or are due to the different origins of the macrophages, blood monocyte-derived compared with bone marrow-derived. In any case, all the above findings underscore the importance of not generalizing fluid-phase macropinocytosis signaling components found in one cell type to all cell types.

Previous reports show that inhibition of the PI3K family with wortmannin or LY294002 inhibits murine M-CSF-differentiated bone marrow-derived macrophage macropinocytosis [26], [27]. In agreement with this, we show inhibition of macropinocytosis, and also LDL uptake and cholesterol accumulation when macrophages are treated with the pan PI3K inhibitors LY294002 or wortmannin, strongly suggesting a role for PI3K in mediating macrophage foam cell formation. Previously, we showed that the class I PI3K isoform gamma contributes to signaling of macropinocytosis in murine GM-CSF-differentiated bone marrow-derived macrophages [47]. However, we show here that no class I PI3K isoform, including gamma, mediated macrophage macropinocytosis of LDL; targeting of individual class I isoforms with isoform-specific inhibitors had no effect on LDL uptake and cholesterol accumulation. We confirmed that class I PI3K beta, delta, and gamma isoforms did not mediate fluid-phase uptake of LDL, because kinase-dead KI macrophages for these isoforms showed similar or greater levels of cholesterol accumulation compared with wild-type macrophages. It is possible that members of Class II or III may mediate macropinocytic uptake of LDL. Because isoform-specific inhibitors and KO animals are currently not available, we were unable to specifically assess the role of these classes of PI3K. Although beyond the scope of this study, the differences in the molecular components mediating macropinocytosis for macrophages differentiated with M-CSF or GM-CSF suggests that specific pharmacologic targeting of macropinocytosis in vivo for these distinct macrophage phenotypes may be possible.

In summary, we demonstrate that murine M-CSF-differentiated bone marrow-derived macrophages take up native LDL by fluid-phase pinocytosis to become macrophage foam cells, and identify fluid-phase pinocytosis signaling components that may be targeted to limit macrophage accumulation of cholesterol accumulation within atherosclerotic plaques.

## Materials and Methods

### Culture of Murine Bone Marrow-derived Macrophages

Male C57BL/6 wild-type and LDL receptor null (LDLR−/−) (C57BL/6 background) mice were obtained from the Jackson Laboratory (Bar Harbor, ME). PI3K-β, γ and δ KI mice were generated as previously described [48], [49]. Femurs and tibias were isolated from mice, and muscle was removed. Both ends of bones were cut with a scissors, and then flushed with 5 ml of RPMI 1640 using a 25-gauge needle to release the contained bone marrow cells. Then, the cells were pressed through a 30-µm nylon mesh filter (Miltenyi, Auburn, CA) and centrifuged at 2000×*g* for 5 min at 4°C. Next, bone marrow cells were resuspended in RPMI 1640 containing 10% FBS, 100 U/ml penicillin, 0.1 mg/ml streptomycin, 2 mM L-glutamine, and 50 ng/ml M-CSF (PeProTech, Rocky Hill, NJ). Bone marrow from one mouse was plated into five 6-well CellBIND culture plates (Corning, Corning, NY). After a 24 h-incubation at 37°C with 5% CO2/95% air, macrophage cultures were rinsed 3 times with 3 ml RPMI 1640 to remove non-adherent cells and cultured with 3 ml of RPMI 1640 containing 10% FBS, 100 U/ml penicillin, 0.1 mg/ml streptomycin, 2 mM L-glutamine, and 50 ng/ml M-CSF, hereafter referred to as complete medium. Cell culture medium was subsequently replaced every 2 days with fresh complete medium. After 1 week of culture, experiments were performed with 1 ml serum-free RPMI 1640 containing 50 ng/ml M-CSF and the indicated additions. M-CSF differentiation of murine bone marrow cells required 1 week to achieve a near uniform culture of macrophages with an elongated shape and numerous vacuoles. All inhibitors were obtained from EMD Millipore (Billerica, MA) except NSC23766 (Caymen Chemical, Ann Arbor, MI), AS605240 (Caymen Chemical), dynamin specific inhibitory peptide (Tocris Bioscience, Bristol, UK), dynamin specific inhibitory peptide control (Tocris Bioscience, Bristol, UK), and Y-27632 (Tocris Bioscience, Bristol, UK). DMSO was used as a vehicle for all inhibitors. All animal procedures were pre-approved by the NHLBI Animal Care and Use Committee.

### Preparation of LDL for Use in Experiments

Human LDL and AcLDL (Intracel, Frederick, MD) was dialyzed at 4°C for 24 h against 1 liter of RPMI 1640 medium containing 100 U/ml penicillin, 0.1 mg/ml streptomycin, and 2 mM L-glutamine, with a medium change at 12 h. Human ^125^I-LDL and ^125^I-AcLDL (BTI, Stoughton, MA) was dialyzed 24 h against RPMI 1640 medium containing 100 U/ml penicillin, 0.1 mg/ml streptomycin, and 2 mM L-glutamine (3 changes, 1 liter/each change). All dialysis was carried out with 10,000 molecular weight cut-off Slide-A-Lyzer cassettes (Pierce, Rockford, IL). After dialysis, lipoproteins were filtered through a 0.45-µm (pore-size) Acrodisc low protein-binding filter (Pall Corporation, Ann Arbor, MI). ^125^I-LDL and ^125^I-AcLDL (BTI, Stoughton, MA) specific activities were adjusted to 2.25×10^−5^ µCi/ng by adding unlabeled LDL and AcLDL, respectively.

### Quantification of Macrophage Cholesterol

Following incubation of macrophage cultures with 1 mg/ml of LDL (unless indicated otherwise) in serum-free RPMI 1640 containing M-CSF (50 ng/ml) and the indicated additions, macrophage cultures were rinsed three times in Dulbecco’s phosphate-buffered saline containing Ca^2+^ and Mg^2+^ (DPBS), lysed with 1 ml/well of ultrapure water, and then detached with a cell scraper. Lipid was isolated using the Folch method [50], and cholesterol was quantified in duplicate aliquots as previously described by Gamble *et al.* [51]. For protein quantification, two 40 µl aliquots of cell lysate were measured using the Lowry method [52] with a BSA standard.

### Visualization of Neutral Lipid

Macrophages were incubated with 4 mg/ml LDL for 24 h, rinsed three times with DPBS, and then fixed 30 min at room temperature with 4% paraformaldehyde in DPBS. Then, the fixed macrophage cultures were rinsed three times with DPBS, and cultures were stained 15 min at room temperature with filtered Oil Red O staining solution. First, Oil Red O stock solution was prepared by dissolving Oil Red O in isopropanol to obtain a 3% (w/v) solution. Next, Oil Red O solution was diluted by adding 2 parts ultrapure water to 3 parts Oil Red O stock solution and then filtered (0.45 µm pore-size). Following Oil Red O staining, macrophage cultures were rinsed three times with ultrapure water, stained two min with Modified Mayer’s hematoxylin QS (Vector Laboratories, Burlingame, CA), rinsed three times with ultrapure water, and then coverslipped with glycerol-gelatin mounting media (Sigma, St. Louis, MO).

### Analysis of ^125^I-LDL and ^125^I-AcLDL Uptake

Macrophages were pretreated with various drugs for one hour and subsequently incubated with the indicated concentration of ^125^I-LDL or ^125^I-AcLDL. ^125^I-LDL or ^125^I-AcLDL macrophage uptake was assessed by measuring cell-associated and degraded ^125^I-lipoprotein according to the method of Goldstein *et al.* [53]. A portion of culture media was centrifuged at 15,000×*g* for 10 min and trichloroacetic acid-soluble organic iodide radioactivity was measured to quantify lipoprotein degradation. Cell-associated ^125^I-lipoprotein was assessed as follows. Macrophages were rinsed first three times with DPBS with 0.2% bovine serum albumin (BSA), followed by three rinses with DPBS containing Ca^2+^ and Mg^2+^, all at 4°C. Macrophages were dissolved overnight in 0.1 N NaOH at 37°C and then assayed for ^125^I radioactivity with a γ counter. ^125^I radioactivity values for wells incubated with ^125^I-lipoprotein without macrophages were subtracted from ^125^I radioactivity values obtained for macrophages incubated with ^125^I-lipoprotein. Values were <1% of cell-associated for ^125^I-lipoprotein. For protein quantification, a small aliquot of cell lysate was measured using the Lowry method [52] with a BSA standard. ^125^I-Lipoprotein uptake is presented as the sum of cell-associated ^125^I- lipoprotein and degraded ^125^I-lipoprotein.

### Time-lapse, Phase-contrast Microscopy

Macrophage cultures were observed by time-lapse, phase-contrast digital video microscopy with a 20X long working distance PanFluor phase-objective lens (0.3 N.A.) mounted on an Olympus L70 inverted microscope. Cultures were maintained in an enclosed LiveCell™ chamber (Pathology Devices, Westminster, MD) containing 5% CO2/95% hydrated air at 37°C. Images were acquired every 10 s for 30 min. The acquired 180 images were converted into digital movies created using IP Lab software (Becton Dickinson, Franklin Lakes, NJ). When viewed at standard rates (i.e., 10 frames/s), movies are 100X real-time.

### Western Blot Analysis

For assessment of Rac1 activation, macrophages were incubated for 1 h with 100 µM NSC23766 or vehicle (DMSO) and then lysed with lysis buffer supplied by the manufacturer’s kit (Pierce, Rockford, IL; catalog number 16118). Then, GTP-bound Rac1 was immunoprecipitated with Pak1 as described by the manufacturer. Lysates were diluted with reducing sample buffer and loaded onto a 4–15% Tris-HCl polyacrylamide gel (BioRad, Hercules, CA). After SDS-PAGE, protein was transferred to nitrocellulose membranes and then incubated overnight at 4°C in Tris-Buffered Saline containing 0.1% Tween (TBST)-20, 0.1% NaN_3_, 3% BSA, and anti-Rac1 mouse monoclonal antibody supplied by the manufacturer’s kit (Pierce, Rockford, IL; catalog number 16118). After incubation with primary antibody, membranes were washed and incubated 1 h with anti-mouse IgG-HRP-conjugate (Santa Cruz Biotechnology, Santa Cruz, CA; catalog number sc-2005) in TBST. Detection of proteins was determined by chemiluminescence using Western Blotting Luminol Reagent (Santa Cruz Biotechnology; catalog number sc-2048).

For assessment of Src-family kinase activation, macrophages were incubated 1 h with 20 µM PP2 or vehicle (DMSO) and then lysed with RIPA Lysis buffer (Santa Cruz Biotechnology; catalog number sc-24948). Lysates were diluted with Laemmli Sample Buffer and loaded onto 4–15% Tris-HCl polyacrylamide gradient gels (BioRad). After SDS-PAGE, protein was transferred to nitrocellulose membranes and then incubated overnight at 4°C in TBST, 3%BSA, and rabbit Phospho-Src Family (Tyr416) Antibody (Cell Signaling, Boston, MA; catalog number 2101). After incubation with primary antibody, membranes were washed and incubated 1 h with anti-rabbit IgG-HRP-conjugate (Santa Cruz Biotechnology; catalog number sc-2004) in TBST. Detection of proteins was determined by chemiluminescence using Western Blotting Luminol Reagent (Santa Cruz Biotechnology; catalog number sc-2048). As a loading control for NSC23766 and PP2 experiments, macrophage lysates were treated with Laemmli Sample Buffer and loaded onto 4–15% Tris-HCl polyacrylamide gradient gels (BioRad). Protein was then transferred to nitrocellulose membranes and incubated overnight at 4°C in TBST, 3%BSA, and mouse anti-GAPDH Antibody (Cell Signaling; catalog number 2118). Membranes were washed and incubated 1 h with anti-rabbit IgG-HRP-conjugate (Santa Cruz Biotechnology; catalog number sc-2004) in TBST and detection of proteins was determined by chemiluminescence using Western Blotting Luminol Reagent.

For tyrosine phosphorylation assessment of the M-CSF receptor, macrophages were incubated 1 hour with 5 µM GW2580 or vehicle (DMSO) and then lysed with RIPA Lysis Buffer (Santa Cruz Biotechnology; catalog number sc-24948) containing Halt Protease and Phosphatase inhibitor (ThermoFisher; catalog number 1861280). The lysates were immunoprecipitated with anti-CSF-1R (Santa Cruz Biotechnology; catalog number sc-692), then protein A agarose beads were added to lysates and incubated overnight. The lysate/bead mixture was then pelleted and resuspended with reducing sample buffer and loaded onto a 4–15% Tris-HCl polyacrylamide gel (BioRad, Hercules, CA). After SDS-PAGE, protein was transferred to nitrocellulose membranes and then incubated overnight at 4°C in Tris-Buffered Saline containing 0.1% Tween-20, 0.1% NaN_3_, 5% BSA, and 4G10 mouse monoclonal antibody (EMD Millipore; catalog number 05–321). After incubation with primary antibody, membranes were washed and incubated 1 h with anti-mouse IgG-HRP-conjugate (Santa Cruz Biotechnology, catalog number sc-2005) in Tris-Buffered Saline containing 0.1% Tween-20, 0.1% NaN_3_, and 5% BSA. Detection of proteins was determined by chemiluminescence using Western Blotting Luminol Reagent (Santa Cruz Biotechnology; catalog number sc-2048).

### Statistical Analysis

Data are presented as the mean ± SEM. The means were determined from three culture wells for each data point. Data was analyzed by the 2-tailed unpaired Student’s t test for statistical significance. A *p* value less than 0.05 was considered significant.

## Supporting Information

Figure S1 **Assessment of M-CSF receptor tyrosine kinase inhibitor activity.** Wild-type macrophages were incubated for 1 h with 5 µM GW2580 or vehicle (DMSO). M-CSF was present in the culture medium before and after GW2580 or vehicle treatment. GW2580-treated and vehicle-treated lysates were probed with M-CSF receptor antibody, GAPDH antibody, or immunoprecipitated with M-CSF receptor antibody and then probed with anti-phosphotyrosine (4G10) for Western blot analysis.
(TIF)

Figure S2 **M-CSF withdrawal decreases macrophage accumulation of LDL-derived cholesterol.** Wild-type macrophages were incubated 6 h with 1 mg/ml LDL without or with M-CSF, and then cholesterol accumulation was assessed.
(TIFF)

Figure S3 **Native LDL induces macrophage foam cell formation.** A. LDLR−/− macrophages were incubated with increasing concentrations of LDL for 24 h and then total cholesterol accumulation was assessed. B and C. LDLR−/− macrophages were incubated without (B) or with 4 mg/ml native LDL (C) for 24 h, and then were stained with Oil Red O to label neutral lipid. Scale bar in B = 75 µm and also applies to C.
(TIF)

Figure S4 **The scavenger receptors CD36 and SRA do not mediate LDL uptake.** Wild-type (WT), CD36 KO (ΔCD36), and SRA KO (ΔSRA) macrophages were incubated 6 h with 200 µg/ml ^125^I-LDL. Incubations were performed in serum-free medium containing 50 ng/ml M-CSF. Uptake values represent the sum of cell-associated and degraded ^125^I-LDL. The range of cell-associated and degraded ^125^I-LDL was 8–9% and 91–92%, respectively. There was no statistical difference between macrophage groups.
(TIF)

Figure S5 **Macropinocytosis in LDLR−/− macrophages** **is inhibited by PI3K and actin polymerization inhibitors.** LDLR−/− macrophages differentiated with M-CSF were pretreated 30 min with the drugs indicated below but without serum and M-CSF. Then, macrophages were treated 30 min with fresh serum-free medium containing M-CSF (50 ng/ml) in the presence of either drug vehicle (A), 4 µg/ml cytochalasin D (B), or 50 µM LY294002 (C). Following treatment, macrophages were examined by phase-contrast microscopy. Arrows indicate macropinosomes. Bar in C = 75 µm and applies to all.
(TIF)

Figure S6 **Assessment of Rac1 and Src-family kinase inhibitor activity.** Wild-type macrophages were incubated for 1 h with 100 µM NSC23766, 20 µM PP2, or vehicle (DMSO). (A) NSC23766-treated and vehicle-treated lysates were probed with GAPDH antibody or immunoprecipitated with Pak1 and then probed with anti-Rac1 for Western blot analysis. (B) PP2-treated and vehicle-treated lysates were probed with phospho Src-family antibody or GAPDH antibody. Incubations were performed in serum-free medium containing 50 ng/ml M-CSF. Phosphorylated Src Family Kinase = p-SFK.
(TIF)

Videos S1 **Macrophage macropinocytosis is M-CSF dependent.** Wild-type macrophages differentiated with M-CSF were pretreated 30 min either with 5 µM GW2580 or vehicle without serum and M-CSF. Then, macrophages were treated 30 min with fresh serum-free medium containing M-CSF (50 ng/ml) in the presence of DMSO vehicle (Video S1) or 5 µM GW2580 (Video S2). During the treatment, macrophage macropinocytosis was visualized by time-lapse, phase-contrast digital microscopy.
(MP4)

Videos S2 **Macrophage macropinocytosis is M-CSF dependent.** Wild-type macrophages differentiated with M-CSF were pretreated 30 min either with 5 µM GW2580 or vehicle without serum and M-CSF. Then, macrophages were treated 30 min with fresh serum-free medium containing M-CSF (50 ng/ml) in the presence of DMSO vehicle (Video S1) or 5 µM GW2580 (Video S2). During the treatment, macrophage macropinocytosis was visualized by time-lapse, phase-contrast digital microscopy.
(MP4)

Videos S3 **M-CSF withdrawal inhibits macrophage macropinocytosis.** Wild-type macrophages differentiated with M-CSF were incubated (Video S3) with or (Video S4) without M-CSF (50 ng/ml) for 30 min, and then macrophage macropinocytosis was visualized by time-lapse, phase-contrast digital microscopy. Incubations were performed in serum-free medium.
(MP4)

Videos S4 **M-CSF withdrawal inhibits macrophage macropinocytosis.** Wild-type macrophages differentiated with M-CSF were incubated (Video S3) with or (Video S4) without M-CSF (50 ng/ml) for 30 min, and then macrophage macropinocytosis was visualized by time-lapse, phase-contrast digital microscopy. Incubations were performed in serum-free medium.
(MP4)

Videos S5 **Macropinocytosis in wild-type macrophages** **is inhibited by PI3K and actin polymerization inhibitors.** Wild-type macrophages differentiated with M-CSF were pretreated 30 min with the drugs indicated below but without serum and M-CSF. Then, macrophages were treated 30 min with fresh serum-free medium containing M-CSF (50 ng/ml) in the presence of either drug vehicle (Video S5), 100 nM wortmannin (Video S6), 4 µg/ml cytochalasin D (Video S7), or 50 µM LY294002 (Video S8). During the treatment, macrophage macropinocytosis was visualized by time-lapse, phase-contrast digital microscopy. Note that LY294002-treatment of macrophages induced small cytoplasmic vacuoles that were not formed by macropinocytosis.
(MP4)

Videos S6 **Macropinocytosis in wild-type macrophages** **is inhibited by PI3K and actin polymerization inhibitors.** Wild-type macrophages differentiated with M-CSF were pretreated 30 min with the drugs indicated below but without serum and M-CSF. Then, macrophages were treated 30 min with fresh serum-free medium containing M-CSF (50 ng/ml) in the presence of either drug vehicle (Video S5), 100 nM wortmannin (Video S6), 4 µg/ml cytochalasin D (Video S7), or 50 µM LY294002 (Video S8). During the treatment, macrophage macropinocytosis was visualized by time-lapse, phase-contrast digital microscopy. Note that LY294002-treatment of macrophages induced small cytoplasmic vacuoles that were not formed by macropinocytosis.
(MP4)

Videos S7 **Macropinocytosis in wild-type macrophages** **is inhibited by PI3K and actin polymerization inhibitors.** Wild-type macrophages differentiated with M-CSF were pretreated 30 min with the drugs indicated below but without serum and M-CSF. Then, macrophages were treated 30 min with fresh serum-free medium containing M-CSF (50 ng/ml) in the presence of either drug vehicle (Video S5), 100 nM wortmannin (Video S6), 4 µg/ml cytochalasin D (Video S7), or 50 µM LY294002 (Video S8). During the treatment, macrophage macropinocytosis was visualized by time-lapse, phase-contrast digital microscopy. Note that LY294002-treatment of macrophages induced small cytoplasmic vacuoles that were not formed by macropinocytosis.
(MP4)

Videos S8 **Macropinocytosis in wild-type macrophages** **is inhibited by PI3K and actin polymerization inhibitors.** Wild-type macrophages differentiated with M-CSF were pretreated 30 min with the drugs indicated below but without serum and M-CSF. Then, macrophages were treated 30 min with fresh serum-free medium containing M-CSF (50 ng/ml) in the presence of either drug vehicle (Video S5), 100 nM wortmannin (Video S6), 4 µg/ml cytochalasin D (Video S7), or 50 µM LY294002 (Video S8). During the treatment, macrophage macropinocytosis was visualized by time-lapse, phase-contrast digital microscopy. Note that LY294002-treatment of macrophages induced small cytoplasmic vacuoles that were not formed by macropinocytosis.
(MP4)

Videos S9 **Effect of GTPase, tubulin, and vacuolar type H(+)-ATPase inhibitors on macrophage macropinocytosis.** Macrophages differentiated with M-CSF were pretreated 30 min with the drugs indicated below but without serum and M-CSF. Then, the macrophages were treated 30 min with fresh serum-free medium containing M-CSF (50 ng/ml) in the presence of either drug vehicle (Video S9), 80 µM dynasore (Video S10), 500 nM bafilomycin A1 (Video S11), 100 µM NSC23766 (Video S12), 10 µM nocodazole (Video S13), or 40 µM Y-27632 (Video S14). During the treatment, macrophage macropinocytosis was visualized by time-lapse, phase-contrast digital microscopy.
(MP4)

Videos S10 **Effect of GTPase, tubulin, and vacuolar type H(+)-ATPase inhibitors on macrophage macropinocytosis.** Macrophages differentiated with M-CSF were pretreated 30 min with the drugs indicated below but without serum and M-CSF. Then, the macrophages were treated 30 min with fresh serum-free medium containing M-CSF (50 ng/ml) in the presence of either drug vehicle (Video S9), 80 µM dynasore (Video S10), 500 nM bafilomycin A1 (Video S11), 100 µM NSC23766 (Video S12), 10 µM nocodazole (Video S13), or 40 µM Y-27632 (Video S14). During the treatment, macrophage macropinocytosis was visualized by time-lapse, phase-contrast digital microscopy.
(MP4)

Videos S11 **Effect of GTPase, tubulin, and vacuolar type H(+)-ATPase inhibitors on macrophage macropinocytosis.** Macrophages differentiated with M-CSF were pretreated 30 min with the drugs indicated below but without serum and M-CSF. Then, the macrophages were treated 30 min with fresh serum-free medium containing M-CSF (50 ng/ml) in the presence of either drug vehicle (Video S9), 80 µM dynasore (Video S10), 500 nM bafilomycin A1 (Video S11), 100 µM NSC23766 (Video S12), 10 µM nocodazole (Video S13), or 40 µM Y-27632 (Video S14). During the treatment, macrophage macropinocytosis was visualized by time-lapse, phase-contrast digital microscopy.
(MP4)

Videos S12 **Effect of GTPase, tubulin, and vacuolar type H(+)-ATPase inhibitors on macrophage macropinocytosis.** Macrophages differentiated with M-CSF were pretreated 30 min with the drugs indicated below but without serum and M-CSF. Then, the macrophages were treated 30 min with fresh serum-free medium containing M-CSF (50 ng/ml) in the presence of either drug vehicle (Video S9), 80 µM dynasore (Video S10), 500 nM bafilomycin A1 (Video S11), 100 µM NSC23766 (Video S12), 10 µM nocodazole (Video S13), or 40 µM Y-27632 (Video S14). During the treatment, macrophage macropinocytosis was visualized by time-lapse, phase-contrast digital microscopy.
(MP4)

Videos S13 **Effect of GTPase, tubulin, and vacuolar type H(+)-ATPase inhibitors on macrophage macropinocytosis.** Macrophages differentiated with M-CSF were pretreated 30 min with the drugs indicated below but without serum and M-CSF. Then, the macrophages were treated 30 min with fresh serum-free medium containing M-CSF (50 ng/ml) in the presence of either drug vehicle (Video S9), 80 µM dynasore (Video S10), 500 nM bafilomycin A1 (Video S11), 100 µM NSC23766 (Video S12), 10 µM nocodazole (Video S13), or 40 µM Y-27632 (Video S14). During the treatment, macrophage macropinocytosis was visualized by time-lapse, phase-contrast digital microscopy.
(MP4)

Videos S14 **Effect of GTPase, tubulin, and vacuolar type H(+)-ATPase inhibitors on macrophage macropinocytosis.** Macrophages differentiated with M-CSF were pretreated 30 min with the drugs indicated below but without serum and M-CSF. Then, the macrophages were treated 30 min with fresh serum-free medium containing M-CSF (50 ng/ml) in the presence of either drug vehicle (Video S9), 80 µM dynasore (Video S10), 500 nM bafilomycin A1 (Video S11), 100 µM NSC23766 (Video S12), 10 µM nocodazole (Video S13), or 40 µM Y-27632 (Video S14). During the treatment, macrophage macropinocytosis was visualized by time-lapse, phase-contrast digital microscopy.
(MP4)

Videos S15 **Dynamin is a mediator of macrophage macropinocytosis.** Wild-type macrophages differentiated with M-CSF were pretreated 30 min either with 20 µM myristoylated dynamin inhibitory peptide (control) or 20 µM dynamin inhibitory peptide without serum and M-CSF. Then, macrophages were treated 30 min with fresh serum-free medium containing M-CSF (50 ng/ml) in the presence of 20 µM myristoylated dynamin inhibitory peptide (control) (Video S15) or 20 µM dynamin inhibitory peptide (Video S16). During the treatment, macrophage macropinocytosis was visualized by time-lapse, phase-contrast digital microscopy.
(MP4)

Videos S16 **Dynamin is a mediator of macrophage macropinocytosis.** Wild-type macrophages differentiated with M-CSF were pretreated 30 min either with 20 µM myristoylated dynamin inhibitory peptide (control) or 20 µM dynamin inhibitory peptide without serum and M-CSF. Then, macrophages were treated 30 min with fresh serum-free medium containing M-CSF (50 ng/ml) in the presence of 20 µM myristoylated dynamin inhibitory peptide (control) (Video S15) or 20 µM dynamin inhibitory peptide (Video S16). During the treatment, macrophage macropinocytosis was visualized by time-lapse, phase-contrast digital microscopy.
(MP4)
