# Supplementary figures and images for: Fluid-Phase Pinocytosis of Native Low Density Lipoprotein Promotes Murine M-CSF Differentiated Macrophage Foam Cell Formation
Source: PLoS One. 2013 Mar 11;8(3):e58054. doi: 10.1371/journal.pone.0058054 (PMC3594233; doi:10.1371/journal.pone.0058054)

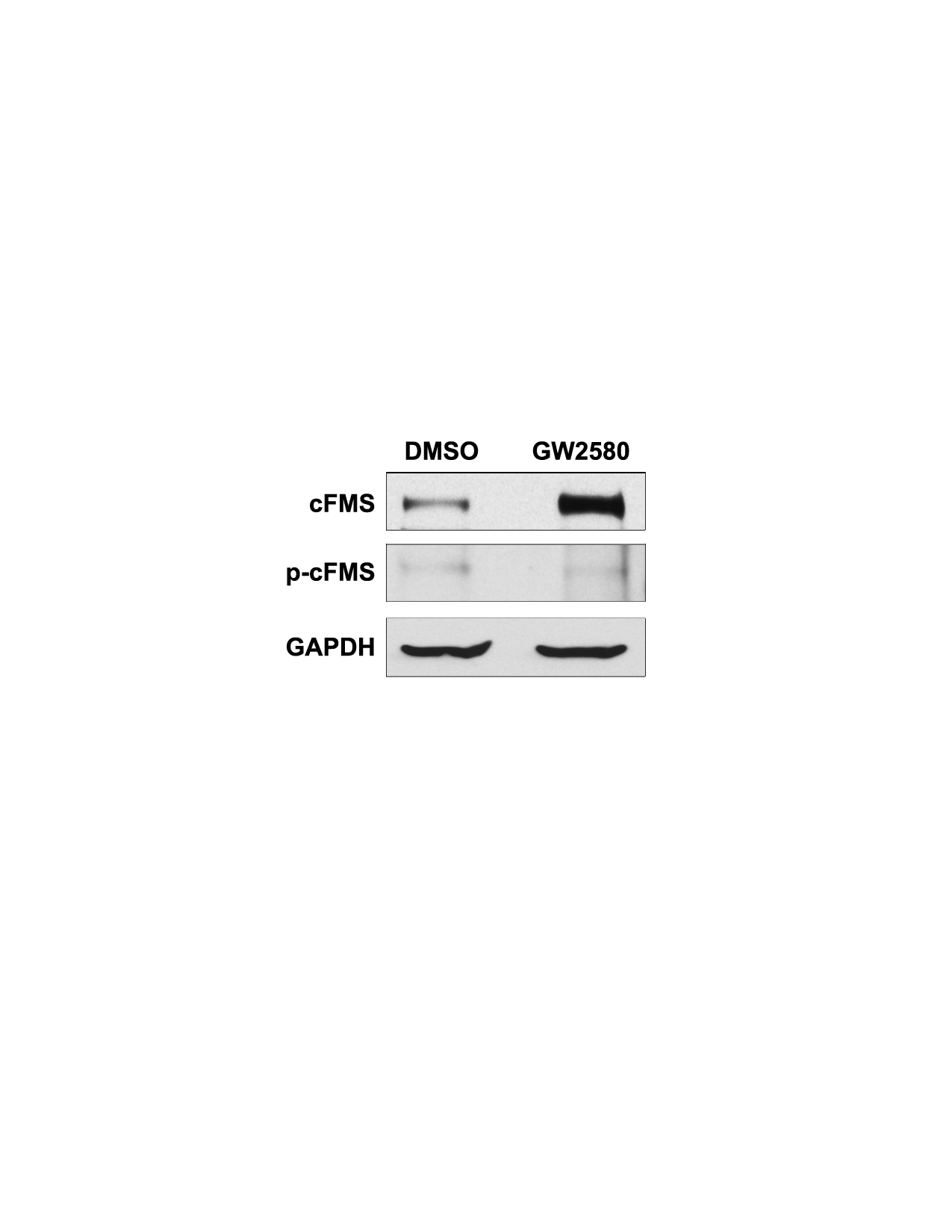

Supplement: Figure S1 — Assessment of M-CSF receptor tyrosine kinase inhibitor activity. Wild-type macrophages were incubated for 1 h with 5 µM GW2580 or vehicle (DMSO). M-CSF was present in the culture medium before and after GW2580 or vehicle treatment. GW2580-treated and vehicle-treated lysates were probed with M-CSF receptor antibody, GAPDH antibody, or immunoprecipitated with M-CSF receptor antibody and then probed with anti-phosphotyrosine (4G10) for Western blot analysis. (TIF) [file pone.0058054.s001.tif]

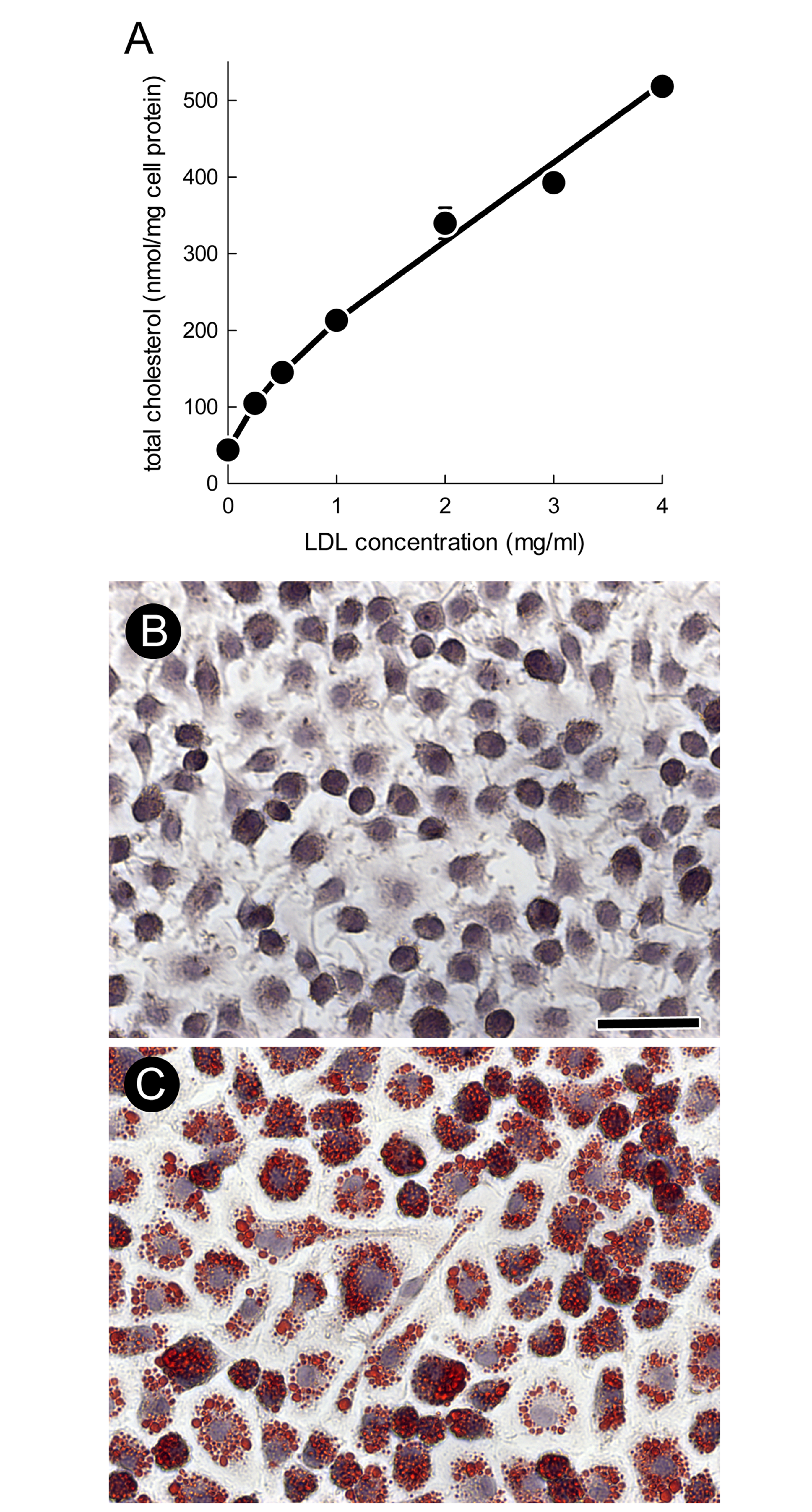

Supplement: Figure S3 — Native LDL induces macrophage foam cell formation. A. LDLR−/− macrophages were incubated with increasing concentrations of LDL for 24 h and then total cholesterol accumulation was assessed. B and C. LDLR−/− macrophages were incubated without (B) or with 4 mg/ml native LDL (C) for 24 h, and then were stained with Oil Red O to label neutral lipid. Scale bar in B = 75 µm and also applies to C. (TIF) [file pone.0058054.s003.tif]

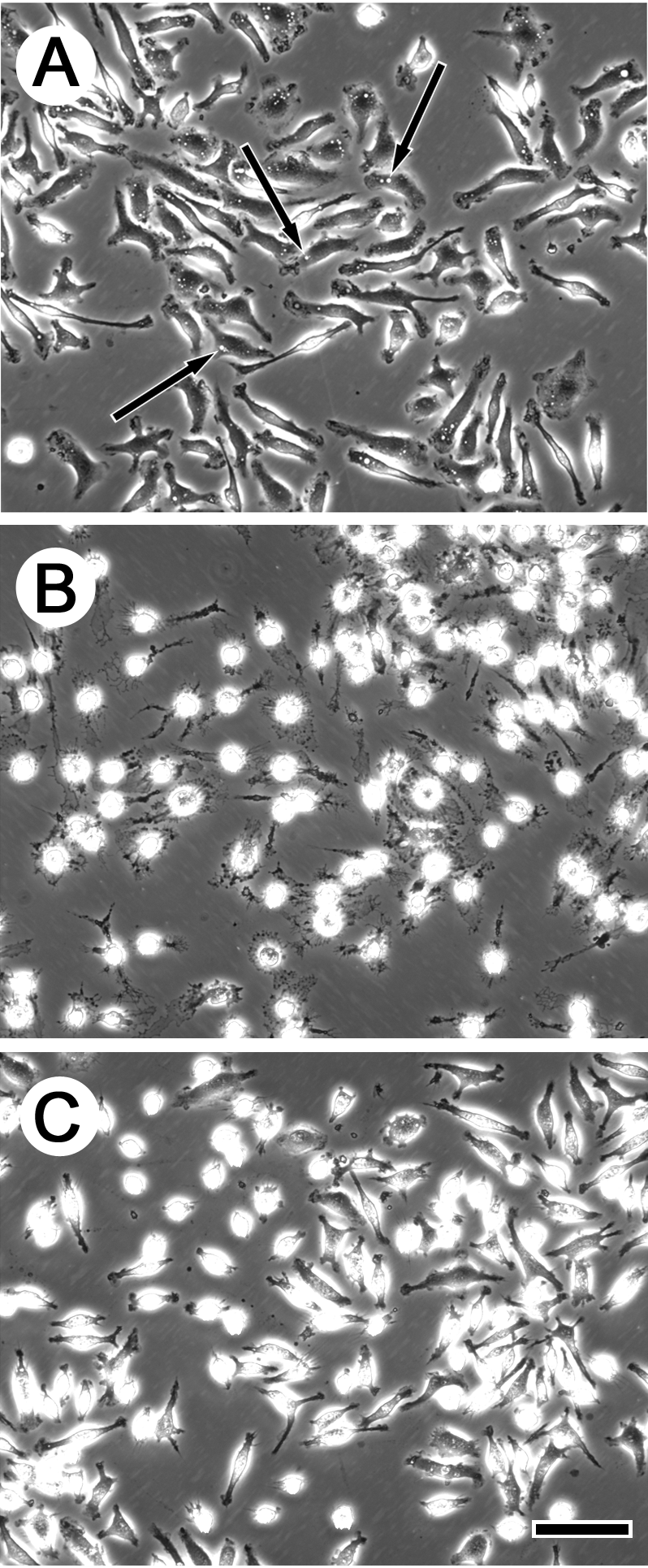

Supplement: Figure S5 — Macropinocytosis in LDLR−/− macrophages is inhibited by PI3K and actin polymerization inhibitors. LDLR−/− macrophages differentiated with M-CSF were pretreated 30 min with the drugs indicated below but without serum and M-CSF. Then, macrophages were treated 30 min with fresh serum-free medium containing M-CSF (50 ng/ml) in the presence of either drug vehicle (A), 4 µg/ml cytochalasin D (B), or 50 µM LY294002 (C). Following treatment, macrophages were examined by phase-contrast microscopy. Arrows indicate macropinosomes. Bar in C = 75 µm and applies to all. (TIF) [file pone.0058054.s005.tif]

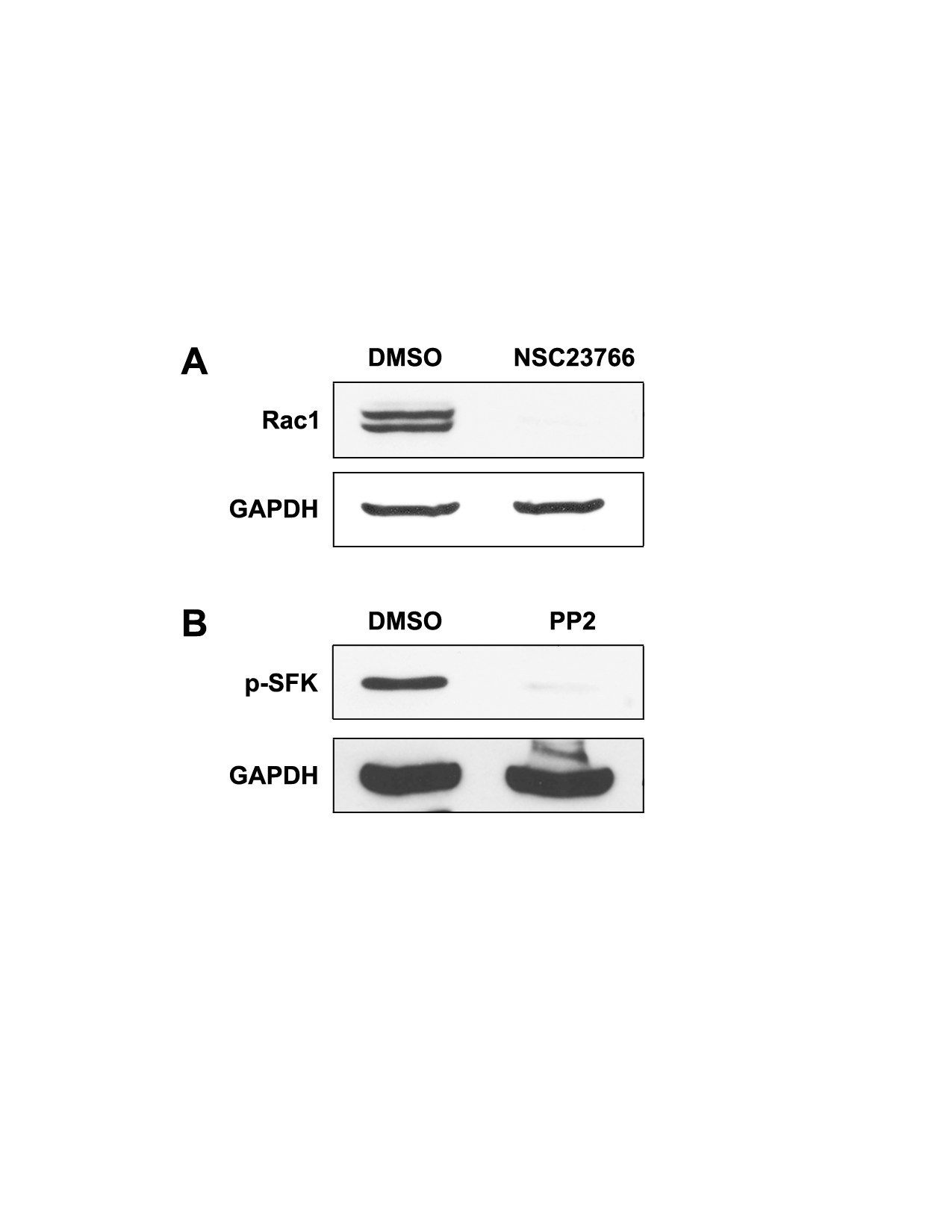

Supplement: Figure S6 — Assessment of Rac1 and Src-family kinase inhibitor activity. Wild-type macrophages were incubated for 1 h with 100 µM NSC23766, 20 µM PP2, or vehicle (DMSO). (A) NSC23766-treated and vehicle-treated lysates were probed with GAPDH antibody or immunoprecipitated with Pak1 and then probed with anti-Rac1 for Western blot analysis. (B) PP2-treated and vehicle-treated lysates were probed with phospho Src-family antibody or GAPDH antibody. Incubations were performed in serum-free medium containing 50 ng/ml M-CSF. Phosphorylated Src Family Kinase = p-SFK. (TIF) [file pone.0058054.s006.tif]
